# The influence of cyclothymic and hyperthymic affective temperaments on glycemic control in patients with type 2 diabetes

**DOI:** 10.1038/s41598-025-90292-w

**Published:** 2025-02-18

**Authors:** Csenge Hargittay, Krisztián Vörös, Ajándék Eőry, Andrea László, Bernadett Márkus, Georgina Szabó, Bálint Tripolszky, Zoltán Rihmer, Xénia Gonda, Péter Torzsa

**Affiliations:** 1 Department of Family Medicine, Faculty of Medicine, Semmelweis University, Budapest, Hungary; 2 Norisana - MVZ Rosenau, Nuremberg, Germany; 3 Doctoral School of Mental Health Sciences, Semmelweis University, Budapest, Hungary; 4 Department of Psychiatry and Psychotherapy, Faculty of Medicine, Semmelweis University, Budapest, Hungary; 5 Department of Clinical Psychology, Faculty of Medicine, Semmelweis University, Budapest, Hungary

**Keywords:** Type 2 diabetes, Affective temperaments, Depressive symptoms, Mediating factors, Primary care, Depression, Type 2 diabetes

## Abstract

Affective temperaments are inherited parts of personality determining mood and activity, affecting the management of somatic conditions. We aimed to investigate the association between affective temperaments, depressive symptoms, and self-care (physical activity, smoking, alcohol consumption), and their effect on glycemic control, among patients with type 2 diabetes (T2DM) in general practice, in a cross-sectional study enrolling 338 consecutive patients from six primary care practices in Hungary. A self-administered questionnaire (history, anthropometric, socioeconomic, laboratory parameters), the Beck Depression Inventory (BDI), the Hamilton Anxiety Scale, and the Temperament Evaluation of Memphis, Pisa, Paris, and San Diego Autoquestionnaire were used. Cyclothymic affective temperament determined HbA1c levels in regression analysis (*p* = 0.002), and the BDI score (*p* = 0.048). In causal mediation analyses, cyclothymic affective temperament was directly associated with higher HbA1c (*p* = 0.008). Hyperthymic affective temperament was indirectly associated with lower HbA1c, mediated by BDI (*p* = 0.034). Depressive, anxious, and irritable affective temperaments, and lifestyle factors were not associated with HbA1c neither in regression nor in mediation analysis as direct or mediating factors. Among primary care patients with T2DM, cyclothymic temperament correlates with worse glycemic control, independently of depressive symptoms. Hyperthymic temperament reduces depressive symptoms, thereby improving glycemic control. Identifying affective temperaments may improve diabetes care.

## Introduction

In somatic medicine, the role of psychological factors in the development, treatment, course, and outcome of illnesses, as well as their role as possible targets for improving the management and well-being of patients is increasingly recognised. Diabetes mellitus is a major health issue in today’s population because of its high prevalence and complications. Diabetes has become one of the 10 leading causes of death, with a significant increase of 70% since 2000^1^. Between 1990 and 2019, the disability-adjusted life years (DALY) attributable to diabetes increased by 24.4%^2^.

Personality and individual psychological characteristics may have an important role in patients’ behaviour. Temperaments have a strong biological basis and are stable parts of the personality, which are inherited and determine the emotional mood, reactivity, and the level of activity. Temperaments are manifested in the early years and are stable throughout life^3^. While several temperamental models were derived on theoretical bases to describe human reactivity in general, the model of affective temperaments was developed based on observations of affective disorder patients and their healthy first-degree relatives, and it was extrapolated to healthy functioning. The affective temperaments (depressive, cyclothymic, hyperthymic, irritable and anxious) are subclinical, trait-related manifestations and are common precursors of affective mood disorders, like unipolar major depression or bipolar (I) and (II) disorders and suicide risk^4^. In addition to their association with psychiatric disorders, numerous studies found a relationship between temperaments and somatic diseases. For example, several studies pointed to string associations between affective temperaments and multiple aspects of cardiovascular diseases including risk, complications and disease course. Robust significant associations have been reported between cyclothymic affective temperament and risk of hypertension^5^, history of acute coronary syndrome and myocardial infarction in hypertensive patients^6^, brachial systolic blood pressure^7^, earlier hypertension onset in women^8^accelerated vascular aging^9^and higher resistant hypertension^10^, while hyperthymic temperament was negatively associated in earlier studies with pulse wave reflection augmentation index^7^, and both of these temperaments played a role in predicting severe coronary artery disease with opposite direction^11,12^. These studies confirmed in new patient populations the pathophysiological role of cyclothymic temperament on the cardiovascular system and the protective role of hypertymic temperament.

Affective temperaments were thus found to play a major role in highly prevalent, chronic, and long-term illnesses, where identification of temperamental factors influencing both risk of development of the disease and its successful long-term treatment would provide an important tool for prevention and better management as well.

Despite being highly prevalent, the relationship between affective temperaments and diabetes mellitus is still elusive and partly controversial. Gois et al. found that patients with excessive depressive temperaments had worse glycemic control^13^. In another study, depressive and cyclothymic temperaments positively correlated with fasting glucose but not with HbA1c values among obese individuals with type 2 diabetes^14^, while both positive^14^and inverse^15^ associations have been reported between anxious temperament and HbA1c.

The aim of our study was:

1. To assess the possible association between affective temperaments and glycemic control among patients with type 2 diabetes in the general practice setting.
2. To investigate whether temperaments affect metabolic control either through depression or by impairing self-care (physical activity, smoking, alcohol consumption).

## Materials and methods

### Patients

A total of 338 adult patients (over 18 years of age) with type 2 diabetes mellitus were included in our study, with the help of accredited general practitioners of the Department of Family Medicine at Semmelweis University, from six primary care practices in Budapest, between September 2018 and February 2020. Consecutive patients attending to the practices were included. The general practitioners were provided with verbal and written information and were consulted if they had any questions. Participation in the study was voluntary. Patients were informed verbally and in written form about the study and gave written informed consent. We excluded patients with type 1 and gestational diabetes mellitus, patients with type 2 diabetes mellitus who had psychiatric outpatient follow-up, or had hospitalization with a psychiatric diagnosis, or cognitive impairment, and patients who were taking antidepressants or antipsychotics. Patients were not rewarded for participating in the study. One patient was excluded because of type 1 diabetes and 5 patients declined to participate, 3 due to lack of time and 2 did not give a reason. The data of the patients were used anonymously.

Our study was approved by the national ethical committee under the number 44677-2/2018/EKU. The research follows the ethical guidelines of the Declaration of Helsinki.

## Measures

The self-report general questionnaire recorded sociodemographic data, addictions, history of suicide attempt and physical activity. The remaining part of the questionnaire was completed by the general practitioners (GPs), based on the patient’s medical records. GPs recorded any co-morbidities, complications, the patient’s medication history and their blood glucose and HbA1c levels. We used the WHO recommended classification for BMI categories (normal BMI: 18.5–24.9 kg/m^2^, overweight: 25.0–29.9 kg/m^2^, obese: ≥ 30.0 kg/m^2^).

### Temperament evaluation of Memphis, Pisa, Paris and San Diego Autoquestionnaire (TEMPS- A)

We used the Temperament Evaluation of Memphis, Pisa, Paris and San Diego Autoquestionnaire (TEMPS-A) to assess affective temperaments on depressive, cyclothymic, hyperthymic, irritable and anxious subscales^16^. The TEMPS-A contains 110 items (109 in the version for males), requiring ‘yes’ (score 1) or ‘no’ (score 0) answers. The mean and standard deviation of each subscale are calculated. The Hungarian version of the questionnaire used in this study was validated in 2006 by Rózsa and coworkers^17^.

### Beck Depression Inventory (BDI) and Hamilton Anxiety Scale (HAM-A)

We chose the Beck Depression Inventory (BDI) to assess the presence and severity of depressive symptoms because it is recommended by the American Diabetes Association for screening symptoms of depression among patients with type 2 diabetes mellitus^18,19^. The BDI is a 21-item, four-point (0–3) self-report questionnaire^20,21^.

Although the American Diabetes Association recommends the 21-item Beck Anxiety Inventory for screening anxiety among patients with type 2 diabetes mellitus, we chose the Hamilton Anxiety Scale (HAM-A), which is still one of the most widely used assessment scales today^22^. According to a study, the specificity and sensitivity of the Beck Anxiety Inventory and the HAM-A were found to be almost identical^23^. During our research, we asked patients for information based on several aspects, so it was important to keep the burden on patients manageable to ensure they did not skip completing the questionnaire. The HAM-A questionnaire was preferred because it is quick to complete and consists of 14 questions in total, as opposed to the 21 questions of the Beck Anxiety Inventory. The HAM-A questionnaire items can be scored from 0 (no symptoms) to 4 (very severe symptoms)^24,25^.

### Statistical analysis

Analyses were conducted using the R statistical software package rms v.6.3 and IBM SPSS Statistical software version 25.0^26,27^. The significance threshold was set at *p* < 0.05. Continuous variables are presented as mean ± standard deviation, categorical variables are presented as count (percentage).

Univariate linear regression analyses were performed to examine the relationship between HbA1c and affective temperaments, BDI, HAM-A, physical activity, smoking, alcohol, age and sex. The analyses used change in TEMPS-A scores, BDI, HAM-A, smoking, physical exercise, alcohol intake, sex and age as predictor variables and HbA1c as dependent variable. After that, causal mediation analysis was applied to examine whether any of the observed variables that significantly predicted HbA1c based on the univariate analysis mediated the effect of affective temperaments on HbA1c. Causal mediation analysis was run with affective temperaments as exposure, HbA1c as response, and any significant predictors of HbA1c as mediator variables.

In mediation analysis, nonparametric bootstrap confidence intervals were calculated with the percentile method^28^. Results are presented as estimated regression coefficients, with 95% confidence intervals. The significance threshold was set at *p*< 0.05. Analyses were conducted using the R statistical software package mediation v.4.5 and IBM SPSS Statistical software version 25.0^27,29^.

## Results

### Characteristics of patients

Descriptive parameters for 338 patients with type 2 diabetes are shown in Table 1.

The mean age of patients was 63.98 ± 11.51 years, 67.2% were 60 years or older, and 61.2% were female. Sociodemographically, nearly one-third had completed only primary education (32.7%), one third had tertiary education (30.1%) and 37.2% had secondary education. Half of the patients lived in the capital city (51.2%). The consumption of alcohol was 35.5%, 13.3% of patients smoked and 21.1% were physically active. More than half of the patients were obese (53.4%) and 33.2% were overweight. 3.1% of patients had a history of suicide attempt. Mean HbA1c was 7.23 ± 1.26% and fasting blood glucose was 8.39 ± 4.11 mmol/l.

The mean BDI score was 8.49 ± 7.65, the mean Hamilton score was 11.08 ± 8.65. The mean depressive affective temperament score was 7.98 ± 3.75 point, cyclothymic 5.8 ± 4.93, hyperthymic 11.3 ± 4.77, irritable 4.91 ± 4.04 and anxious 7.92 ± 6.43.

**Table 1:** Basic description of the sample in primary care, Hungary (2018–2020, *n* = 338).

Total (*n*)	338
Age (years)	63.98 ± 11.51
Gender (female/male)	207 (61. 2%)/131 (38.8%)
Education	
Elementary	110 (32.7%)
Secondary	125 (37.2%)
Tertiary	101 (30.1%)
Residence	
Capital city	173 (51.2%)
City	142 (42.0%)
Village	23 (6.8%)
Alcohol consumption	120 (35.5%)
Smokers	45 (13.3%)
Physical exercise	71 (21.1%)
Suicide attempt	10 (3.1%)
Glycated hemoglobin (%)	7.23 ± 1.26
Blood glucose (mmol/l)	8.39 ± 4.11
BMI (kg/m^2^)	31.09 ± 5.91
Normal BMI (18.5–24.9 kg/m^2^)	45 (13.4%)
Overweight (25–29.9 kg/m^2^)	112 (33.2%)
Obese (≥ 30 kg/m^2^)	180 (53.4%)
BDI (score)	8.49 ± 7.65
HAM-A (score)	11.08 ± 8.65
Depressive temperament (score)	7.98 ± 3.75
Cyclothymic temperament (score)	5.8 ± 4.93
Hyperthymic temperament (score)	11.3 ± 4.77
Irritable temperament (score)	4.91 ± 4.04
Anxious temperament (score)	7.92 ± 6.43

### Regression analysis

Univariate linear regression analyses showed that cyclothymic affective temperament and BDI were associated with HbA1c. A 1-point increase in cyclothymic score was associated with a 0.043% increase in HbA1c (*p* = 0.002), while a 1-point increase in BDI score was associated with a 0.017% increase in HbA1c (*p* = 0.048). Depressive, anxious, irritable, and hyperthymic affective temperaments were not associated directly with HbA1c. Lifestyle factors, such as smoking, physical activity, and alcohol intake were not predicting HbA1c. Table 2. presents the results of the regression analyses.

**Table 2:** Results of univariate regression analyses of possible predictors of HbA1c (*n* = 338).

Variable	Effect size (β)	95% CI		*p*	*R*-squared
Cyclothymic temperament	**0.043**	**0.016**	**0.069**	**0.002**	**0.029**
Depressive temperament	0.025	−0.010	0.060	0.167	0.006
Anxious temperament	0.015	−0.005	0.036	0.148	0.006
Irritable temperament	0.030	−0.001	0.062	0.060	0.010
Hyperthymic temperament	0.003	−0.025	0.030	0.850	0.000
BDI	**0.017**	**0.000**	**0.035**	**0.048**	**0.012**
HAM-A	0.004	−0.011	0.020	0.565	0.001
Physical exercise (yes)	−0.121	−0.349	0.108	0.301	0.003
Smoking (yes)	−0.079	−0.353	0.196	0.575	0.001
Alcohol consumption (yes)	−0.090	−0.285	0.104	0.362	0.002
Gender (female)	−0.042	−0.234	0.149	0.664	0.001
Age	−0.008	−0.020	0.003	0.156	0.006

CI: confidence interval, BDI: Beck Depression Inventory, HAM-A: Hamilton Anxiety Scale. The bold values in the table represent significant findings.

### Causal mediation analysis

**Fig. 1:**
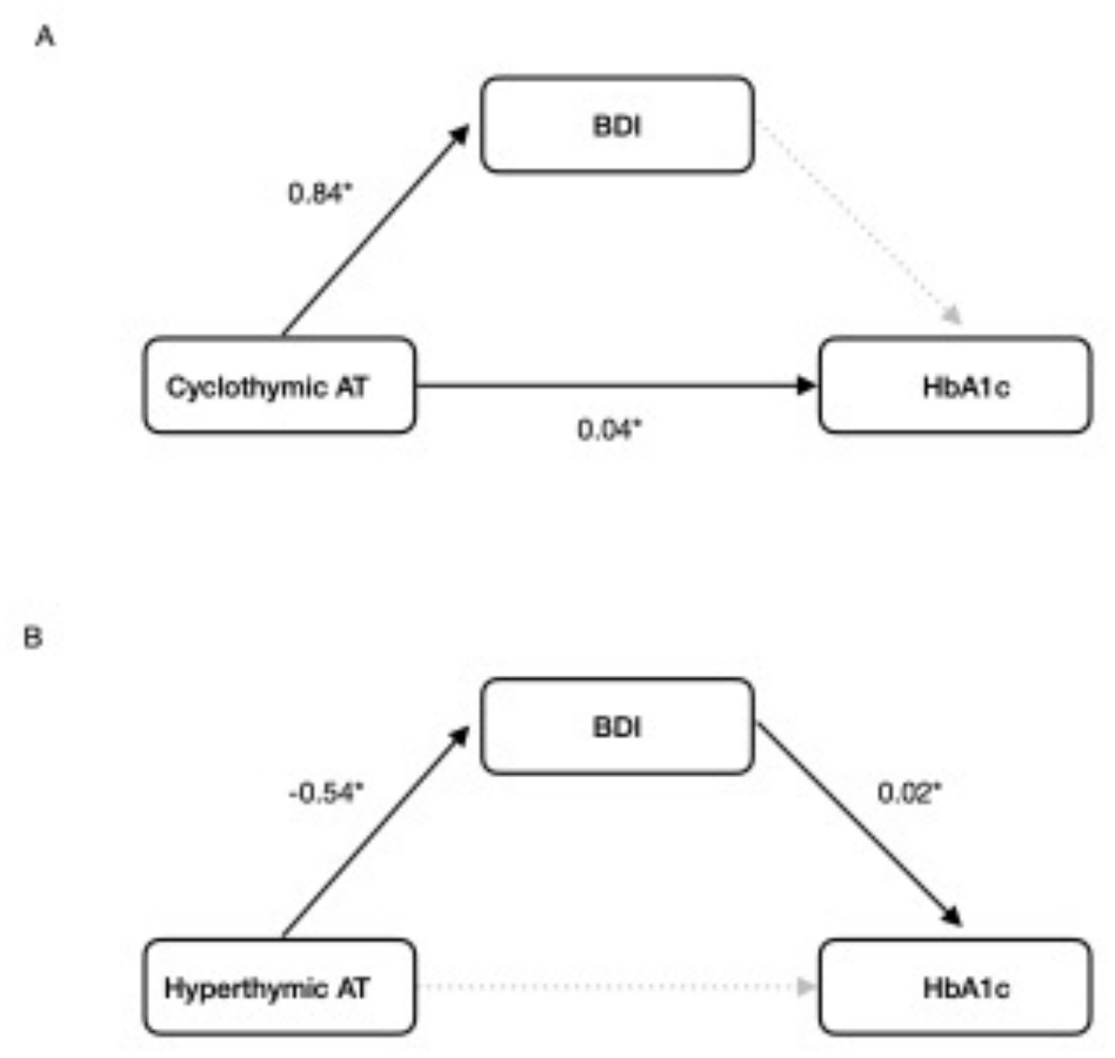
Mediation analysis results. (**A**) Direct effect of cyclothymic affective temperament on HbA1c. (**B**) Indirect effect of hyperthymic affective temperament on HbA1c, mediated by BDI. Key: AT: affective temperament. *indicates significant values.

Since besides affective temperaments, only BDI was a significant predictor of HbA1c based on univariate regression analyses, causal mediation analysis was run with affective temperaments as exposure, BDI as mediator and HbA1c as response variables. Based on causal mediation analyses, a 1-point higher cyclothymic affective temperament score was directly associated with a 0.04% higher HbA1c score (*p* = 0.008), with the effect not mediated by BDI, while a 1-point higher hyperthymic affective temperament score was indirectly associated with a 0.011% lower HbA1c score, mediated by BDI (*p* = 0.034) (Fig. 1). Depressive, anxious, and irritable affective temperaments were not associated with HbA1c neither directly nor indirectly. Table 3. presents the results of the causal mediation analyses.

**Table 3:** Results of mediation analysis of affective temperaments on HbA1c with BDI as mediator (*n* = 338).

	Mediation effect				Direct effect			
Exposure	Effect size estimate	95% CI		*p*	Effect size estimate	95% CI		*p*
Cyclothymic temperament	0.003	−0.015	0.018	0.720	**0.040**	**0.009**	**0.074**	**0.008**
Depressive temperament	0.018	−0.003	0.040	0.100	0.006	−0.036	0.049	0.760
Anxious temperament	0.011	−0.002	0.025	0.100	0.004	−0.022	0.030	0.760
Irritable temperament	0.010	−0.005	0.025	0.182	0.020	−0.014	0.052	0.270
Hyperthymic temperament	**−0.011**	**−0.022**	**−0.001**	**0.034**	0.014	−0.025	0.049	0.448

CI: confidence interval, BDI: Beck Depression Inventory. The bold values in the table represent significant findings.

## Discussion

In our study focusing on the association between affective temperaments and glycemic control, we found a significant direct association between cyclothymic affective temperament and HbA1c. Furthermore, mediation analyses revealed that the effect of cyclothymic affective temperament on HbA1C (a temperament score 1 point higher was directly associated with an HbA1c score increase of 0.04% (*p*= 0.008)), was not mediated by BDI. On the other hand, while hyperthymic temperament was not directly associated with HbA1c, we found a significant effect mediated by BDI. Our results suggest that cyclothymic temperament directly affects glycemic control independently of depression (although depression had a significant direct effect on glycemic control, and cyclothymic temperament was also significantly associated with depression scores), while hyperthymic temperament exerts its effect via reduced BDI scores and thus lowering their deleterious effect on glycemic control, as already demonstrated in our previous study^30^. More severe depressive symptoms may impair self-care in people with diabetes^31^(diet, physical activity and medication adherence), which plays an important role in metabolic control^32^. Therefore, it is important to understand the complex association between affective temperaments and depression in their relationship with diabetes, to identify intervention targets that could improve disease management and glycemic control.

Previous studies reported significant results on the relationship between affective temperaments and diabetes mellitus confirming their role, however, results concerning the specific temperaments were divergent. Gois et al. examined the association between affective temperaments and metabolic control among patients with type 2 diabetes. In their relatively small sample (90 patients) they chose to investigate only anxious and depressive temperaments, making it difficult to compare to our study where all temperaments were included^13^. Another study investigated the relationship between affective temperaments and glycemic control among 185 obese patients with type 2 diabetes mellitus. Beside HbA1c the researchers used the less reliable fasting glucose as dependent variable as well. The reported correlation between depressive, cyclothymic and anxious temperaments and fasting glucose should be interpreted cautiously. However, they found a significant relationship between HbA1c values and anxious temperament^14^.

This finding is conflicting with the results of Hall et al., who reported an inverse association between anxious temperament and HbA1c values among newly diagnosed patients with type 2 diabetes^15^. Individual differences in anxious temperament were assessed using the Behavioral Inhibition Scale (BIS), the Behavioral Inhibition/Behavioral Approach Scales (BIS-BAS) questionnaire and other temperament types were not measured. Omitting other temperaments in these studies might have lead to attaching too much importance to anxious and depressive temperaments, while glossing over the effect of other temperaments.

While sample size^13^, questionnaire used, and not including all temperaments^13,15^probably affected findings, some other factors, including gender ratio or age of the patients^33^ could also explain contradicting results.

Cyclothymic temperament is associated with instability, both in terms of activity and mood, unstable self-esteem, socialisation and energy levels as well as rapid mood swings^34,35^, which may significantly impact therapeutic adherence both in terms of medication, diet, and other recommended lifestyle changes, independently of depressed mood. However, other psychological and behavioural factors, as well as biological, neurobiological and even genetic contributors may play a role in the association between cyclothymic temperament and worse glycaemic control, which warrants further studies to improve our understanding and put findings to clinical use.

Variability in personality or temperament can affect lifestyle choices, self-care, adherence to treatment, and other factors that can affect glycemic control^36^. Modifiable risk factors such as exercise, smoking and alcohol consumption may play a role in the relationship between cyclothymic temperament and glycemic control. In our study, we did not find a significant association between lifestyle factors and glycemic control, and thus they are not considered as mediators, although the literature suggests that they may be involved in mediation. Individuals with cyclothymic temperament are known to smoke more often and consume alcohol at higher rates^37,38^. Cyclothymic temperament can also affect glucose metabolism through adherence to therapy. Yamamoto et al. investigated the link between affective temperaments and poor glycemic control due to insufficient self-care among 77 outpatients with type 2 diabetes mellitus. Significant associations were found between poor glycemic control and stress-induced overeating and poor medication adherence. Furthermore, the cyclothymic temperament was significantly associated with stress-induced overeating and poor medication adherence. Path analysis found a possible causal link between cyclothymic temperament and poor glycemic control through disordered eating and poor medication adherence^39^. In contrast, Shamsi et al. found a relationship between irritable temperament and medication compliance in 207 patients with type 2 diabetes. Irritable temperament has a negative effect on medication compliance and through this, on glycemic control^40^. Szabó et al. recent meta-analysis (9 studies, *n*= 1138) also supports the results of the above studies, as they found that cyclothymic, depressive and irritable temperaments may influence adherence to therapy^41^.

Another important finding of our study is the association between hyperthymic temperament and glycemic control mediated by depression scores. Hyperthymic temperament, associated with a more stable mood, a positive and optimistic attitude and increased energy levels as well as easier and better adaptation to stress^34,42^and has a beneficial role on glucose metabolism by reducing depressive symptoms. Our finding is also in part confirms previous results reporting a negative correlation with HbA1c among obese patients with type 2 diabetes mellitus^14^. It must be noted, however, that our study also managed to show that the relationship is not direct but is rather mediated through decreased depression. Understanding by which other mechanisms hyperthymic temperament is protective against impaired glycemic control and how they can be exploited in the management of these patients is also warranting further studies.

Our research has some limitations, which should always be taken into account when interpreting the results and applying them more widely. We used validated questionnaires, but we could not exclude misunderstandings and errors by patients. In addition, the cross-sectional nature of the study does not allow us to explore causal relationships. The lack of a healthy, non-diabetic control group also limits the generalizability of our results. The subjects in the study were all of Caucasian origin, and as race-specific differences may be present in the affective temperament patterns, this may also be a limitation.

## Conclusion

Among patients with type 2 diabetes mellitus in primary care, cyclothymic temperament correlates with worse glycemic control, with the effect not mediated by BDI, but through a different route. Further studies are needed to explore which factors may be involved. Another important finding of our study is that hyperthymic temperament reduces depressive symptoms as expected, thereby improving glycemic control. Altogether, our results suggest that screening for temperamental and personality factors may help predict treatment outcome in Type 2 diabetes patients and also identify those, where limited adherence may compromise efficacy.

## Data Availability

Raw data are available upon request to the corresponding author.
